# Influenza–Host Interplay and Strategies for Universal Vaccine Development

**DOI:** 10.3390/vaccines8030548

**Published:** 2020-09-20

**Authors:** Hye Suk Hwang, Mincheol Chang, Yoong Ahm Kim

**Affiliations:** 1 Alan G. MacDiarmid Energy Research Institute, Chonnam National University, Gwangju 61186, Korea; hshwang33@gmail.com; 2 Department of Polymer Engineering, Graduate School, Chonnam National University, Gwangju 61186, Korea; 3 School of Polymer Science and Engineering, Chonnam National University, Gwangju 61186, Korea

**Keywords:** influenza A virus, innate immune response, adaptive immune response, immunopathology, universal influenza vaccine

## Abstract

Influenza is an annual epidemic and an occasional pandemic caused by pathogens that are responsible for infectious respiratory disease. Humans are highly susceptible to the infection mediated by influenza A viruses (IAV). The entry of the virus is mediated by the influenza virus hemagglutinin (HA) glycoprotein that binds to the cellular sialic acid receptors and facilitates the fusion of the viral membrane with the endosomal membrane. During IAV infection, virus-derived pathogen-associated molecular patterns (PAMPs) are recognized by host intracellular specific sensors including toll-like receptors (TLRs), C-type lectin receptors, retinoic acid-inducible gene-I (RIG-I)-like receptors (RLRs), and nucleotide-binding oligomerization domain (NOD)-like receptors (NLRs) either on the cell surface or intracellularly in endosomes. Herein, we comprehensively review the current knowledge available on the entry of the influenza virus into host cells and the molecular details of the influenza virus–host interface. We also highlight certain strategies for the development of universal influenza vaccines.

## 1. Introduction

**Figure 1:**
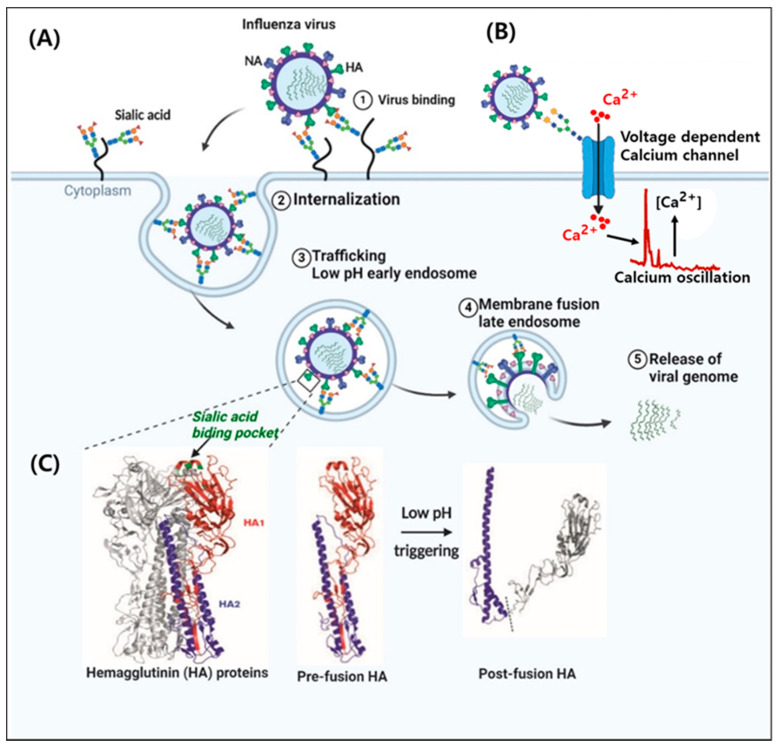
Influenza A virus endocytosis and hemagglutinin proteins (HA) conformational change. (**A**) Process of the entry of influenza virus into host cell. The virus binds to sialic acid-containing proteins on the cell surface receptors by association with the viral hemagglutinin proteins (HA1, HA2). HAs also bind to the sialic acid-containing Ca^2+^ channel to trigger intracellular Ca^2+^ oscillations. The virus is then internalized by endocytosis. Acidification of the endosome causes a conformational change in the HA proteins that leads to a fusion between the viral membrane and the endosomal membrane. This allows the escape of the viral RNA and proteins into the cytoplasm. (**B**) Structure of the HA of IAV. The trimeric complex of HA is shown with one monomer highlighted in color (HA1; red, HA2; blue, and the receptor binding pocket; green). (**C**) The pre- and post-fusion conformations of HA [4]. This figure was created using BioRender (Toronto, ON, Canada).

Influenza A viruses (IAVs) belong to the family *Orthomyxoviridae*, which exhibits 8-segmented, single-stranded, and negative-sense ribonucleic acid genome encoding 11 viral genes, namely, hemagglutinin (HA), neuraminidase (NA), two matrix proteins (M1 and M2), a nonstructural protein (NS1), a nuclear export protein (NS2), a nucleoprotein (NP), an RNA polymerase acidic protein (PA), a polymerase basic protein 1 (PB1), and a polymerase basic protein 2 (PB2), and polymerase basic protein 1-F2 (PB1-F2) [1]. IAVs naturally infect a variety of birds and mammals including humans, while influenza B and C viruses are limited to humans [2]. The high mutation rates and frequent reassortment of the RNA genome in IAVs contribute to the formation of various types of HA and NA antigens [3]. Antigenic drift, which is characterized by small changes in the protein structure of IAVs, occurs frequently and enables the virus to cause repetitive seasonal influenza outbreaks [4,5]. Antigenic shift, also characterized by major changes in the HA glycoprotein, is caused by rearrangement of genomes involving different influenza A subtypes and causes large global pandemics [6,7]. In general, 16 HA subtypes and 9 NA subtypes are known [8]; however, only the H1, H2, and H3 HA subtypes and N1 and N2 NA subtypes have circulated extensively among humans over the past century [9]. IAV surface glycoproteins, namely, HA (receptor binding) and NA (receptor destroying) should balance their functions to allow pathogen attachment to host cell receptors and subsequent release of new virions from the host cell at the end of the viral life cycle [3]. IAV entry is a dynamic multistep process including five individual steps listed as follows: ① attachment to target cell receptors, ② internalization into cellular compartments, ③ endosomal trafficking to the cytoplasmic region, ④ the fusion of viral and endosomal membranes and uncoating, and ⑤ import of the viral genome into the nucleus (Figure 1A) [10].

Influenza vaccines have been in use for over 70 years. However, some promising preclinical data with respect to an influenza universal vaccine have not yet been developed to the point of human clinical testing [11]. Prior immunity to influenza may influence vaccine efficacy. Yet, considering the importance of prior immunity in vaccination, a fundamental understanding of immunity to influenza has been overlooked. Few studies have assessed immune correlates derived from asymptomatic influenza virus infections [11]. Vaccine scientists should consider the correlation of the fundamental understanding of influenza immunity with protection that has been demonstrated across the spectrum of severity from asymptomatic infection to severe illness leading to hospitalization. Accordingly, we review the mechanisms by which the virus manages to successfully enter the host target cells and transport its genetic material to the nucleus. Furthermore, we discuss the innate immune sensors of host cells that play an important role in the recognition of virus-derived PAMPs and the signaling mechanisms that are induced by host–virus interaction in numerous innate and adaptive immune cells to defend the host. In particular, we highlight the recent developments on the universal influenza vaccine approaches that are envisioned to play a potential role in the future of influenza prevention.

## 2. Molecular and Cellular Interaction at the Virus–Host Interface

Upon viral infection, HA, the major surface glycoprotein of influenza virus, binds to the sialylated receptors of the host cell surface glycoproteins. Sialic acid is the distal residue on the oligosaccharide chains of glycoproteins and glycolipids on the host cell surface [12]. Recently, Fujioka et al. demonstrated that the voltage-dependent Ca^2+^ channel Cav1.2 acting as a sialylated host cell surface receptor binds to IAV HA and contributes to IAV entry and replication (Figure 1B) [13]. IAV infection elevates oscillations in the cytosolic calcium concentration of host cells. Human-adapted HAs preferentially recognize α2,6-sialic acid, whereas avian HAs have a binding preference for α2,3-sialic acid [14]. This difference is an important factor contributing to the species-specific tropism of influenza viruses, which has likely evolved in response to ancient retroviruses [15]. A single amino acid mutation in the receptor-binding domain of HA can switch the specificity from human- to avian-type in an H3N2 virus after a single passage in eggs [16] and from avian- to transmissible human-type in an H1N1 virus after a single passage in ferrets [17]. A single G225D mutation in the HA of H6N1 remarkably shifts receptor specificity from avian to human type [18]. However, H5N1 required several mutations to acquire human-type receptor specificity and respiratory droplet transmission between ferrets [19,20,21].

HA binding to sialylated receptors does not always result in the internalization of the virus into the host cell, despite the wide acceptance of sialic acid as the main receptor for IAVs. After initial attachment to host cells, IAVs enter the host cells by receptor-mediated endocytosis via DC-SIGN/L-SIGN [22]. DC-SIGN/L-SIGN recognizes glycans expressed in the viral HA and NA glycoproteins [23]. Furthermore, calcium-dependent C-type lectins [24,25], annexin V [26], and 6-sulfo sialyl Lewis X [27] have been proposed as additional receptors required for completion of IAV entry [10]. IAV uses two entry systems: clathrin- and caveolin- dependent endocytosis and macropinocytosis [10]. During clathrin-mediated endocytosis, virions are internalized into the cytoplasm via a dynamin-dependent route, while the virions internalized by macropinocytosis are transferred via a dynamin-independent route [10]. De Conto et al. explained that the pathogen entry route is likely to be cell-type dependent [28]. Further studies have shown that filamentous IAVs prefer to be internalized via micropinocytosis [29] and require the acidification of endosomes, making dynamin dispensable [30]. After an initial association with the virus–host interaction, microtubule transport machinery facilitates the movement of early endosomes containing IAV virions by forming a physical bridge between viruses and their replication and assembly sites [31].

At the entry sites, IAVs encounter highly glycosylated mucosal defense proteins. NA facilitates virion release from infected cells via sialidase activity, thereby allowing virions to successfully reach the target host cells [32,33]. Additionally, NA cooperates with HA to enable IAVs to crawl and glide on cell surfaces, thereby enhancing viral fusion with the host cells [34]. Furthermore, the NA of IAVs cleaves the terminal sialic acid from viral and cellular glycoconjugates to support multiple infection cycles by the release of newly assembled viral particles from infected cells. This reaction prevents HA mediated aggregation and stops the binding of new virions to the surface of the dying host cell, thereby enabling the efficient release of progeny viral particles and further dissemination toward new cell targets [35]. NA also exhibits a secondary sialic acid binding site and Ca^2+^-ion binding site as well as enzymatic active sites [36]. The stalk domain of NAs includes at least one cysteine residue and a potential glycosylation site. The cysteine residue(s) may assist with tetramer stabilization by enabling the formation of disulfide bonds between the pairs of cysteine residues situated on neighboring monomeric NA units [35,37]. The carbohydrate side chains within the stalk are thought to contribute further to the stability of the tetramer and to the enzymatic functionality of NA [38].

The efficient release of viral genomes requires sequential exposure to the pH of both early and late endosomes along with significant structural rearrangement [39]. Influenza virus matrix protein 1 (M1) forms a coat inside the viral envelope and simultaneously binds to the membrane and RNA [40]. Reversible modification of both HA and the viral lumen occurs in early endosome at pH 7.5–6.0, whereas irreversible M1 dissociation and the pre- to post-fusion conformational changes of HA appear in late endosomes at pH < 6.0 [10,39]. The transient exposure to acidic pH leads HA from the pre-fusion conformation to the post-fusion coiled-coil conformation (Figure 1C). The M2 proton-selective viroporin is an integral membrane protein that oligomerizes to form channels in the membrane [41]. Following the fusion of the virus and the host cell, the opening of the M2 proton channel triggers the acidification of the viral lumen at pH 6.0 and offers more stable circumstances for facilitating the uncoating of the virus and the unloading of viral ribonucleoproteins (RNPs) into the host cytoplasm [10,39]. When vRNPs are released in the cytoplasm, histone deacetylase 6 (HDAC6) is involved in viral transport and fusion and the release of viral components by the modulation of cytoskeletons and plasma membrane dynamics [42,43]. Recently, it was reported that HDAC6 is also related to the suppression of IAV RNA polymerase activity via deacetylation of PA protein [44] and RIG-1 to recognize viral RNA [45].

## 3. Pathogen-Recognition by Host Cells

**Figure 2:**
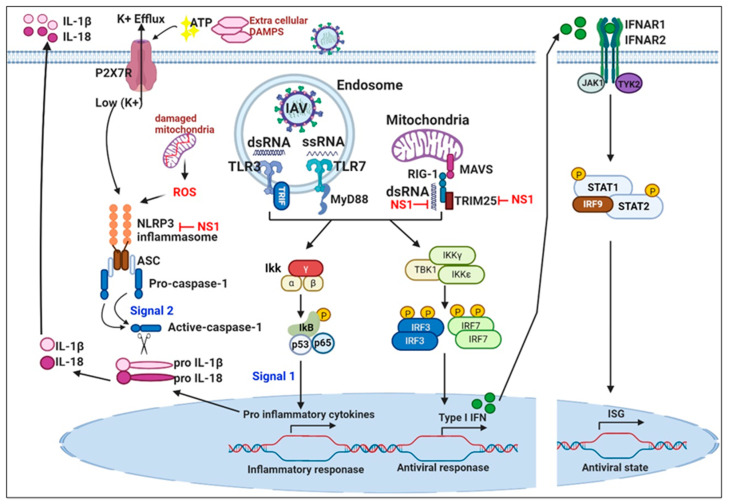
The intracellular cytoplasmic pattern-recognition receptor RIG-I is essential for the control of RNA virus infection. Upon IAV recognition, RIG-I recruits the adaptor MAVS protein to activate the IKKα–IKKβ and TBK1–IKKϵ complexes, which are responsible for the activation of the IRF 3 and IRF7 transcription factors. These transcription factors then translocate into the nucleus and cooperatively induce IRF dependent type I IFNs and NF-κB (nuclear factor kappa-light-chain-enhancer of activated B cells) dependent pro-inflammatory cytokines and chemokines. This is followed by the binding of the IFNAR1 and IFNAR2 to their cognate receptor, which leads to the transcriptional activation of ISGs by the JAK/STAT signaling pathway. The products of ISGs are key factors limiting pathogen spreading. Moreover, ssRNA from IAVs can prime the inflammasome by activating a TLR inducing NF-κB activation and the expression of NLRP3, ASC, and preforms of IL-18 and IL-1β. A second activation signal is provided by the oligomerization of the NLRP3 complex and recruitment of ASC and procaspase-1, allowing the processing and cleavage of pro-IL-1β and pro-IL-18 precursors into their bioactive mature forms (IL-18 and IL-1β). NLRP3 can be activated by imbalances in potassium ion concentration in intracellular vesicles through the ATP-gated P2 × 7 channel and responses of mitochondrial reactive oxygen species. This figure was created using BioRender. RIG-1;retinoic acid-inducible gene-1, MAVS; mitochondrial antiviral signaling adaptor, IKKα; nuclear factor of kappa light polypeptide gene enhancer in B-cells inhibitor (IκB) kinase α, IKKβ; IκB kinase β, IKKϵ; IκB kinase ϵ, TBK1; TRAF family member-associated NF-kappa-B activator (TANK)-binding kinase 1, IRF; interferon-regulatory factors, IFNs; Interferons, NF-κB; Nuclear Factor kappa-light-chain-enhancer of activated B cells, IFNAR1; Interferon Alpha And Beta Receptor Subunit 1, IFNAR2; Interferon Alpha And Beta Receptor Subunit 2, ISGs; interferon-stimulated gene, JAK/STAT; Janus kinase (JAK)/signal transducer and activator of transcription (STAT), ssRNA; single stranded RNA, TLR; Toll like receptor, NLRP3; nucleoside oligomerization domain (NOD), leucine-rich repeat (LRR), and pyrin domain (PYD) domain-containing protein 3, ASC; Apoptosis-associated speck-like protein containing a CARD, IL-18; Interleukin 18, IL-1β; Interleukin 1β, ATP-gated P2X7; Adenosine triphosphate (ATP)-gated purinergic P2X7 receptor.

To efficiently facilitate viral replication and spreading, IAVs have demonstrated multiple strategies to circumvent the potent antiviral activities of the host signaling cascade mediated by the interferon (IFN) and cytokine systems (PRR detection, intermediate signaling molecule activation, transcription factor activation, and the actions of antiviral proteins) [46]. Upon IAV infection, viral pathogen-associated molecular patterns (PAMPs) are recognized by host pattern recognition receptors (PRRs). PAMPs are unique features present in viruses, but not in the host cell, thereby allowing the cells to identify infectious non-self-molecules for eliciting an immune response against infection. Several types of PRRs are known that sense viral infection, including toll-like receptors (TLRs), RIG-I-like receptors (RLRs), and NOD-like receptors (NLRs). Activation of such PRRs triggers signaling cascades via adaptor proteins, such as the mitochondrial antiviral signaling adaptor (MAVS) and the stimulator of interferon genes (STING), followed by the activation of kinases and transcription factors that induce the expression of type I and III IFNs (Figure 2) [46].

### 3.1. Toll-Like Receptors (TLRs)

TLRs are an essential arm of antigen-presenting cells (i.e., macrophages and plasmacytoid dendritic cells) that respond to virus infection by inducing innate immune responses. TLRs are transmembrane proteins expressed by multiple cell types (monocytes and DCs) and are located on either the cell surface (TLR1, 2, 4, and 5), or on cytoplasmic structures such as endosomes (TLR3, 7, 8, and 9). TLR7 is known to recognize IAVs. IFN-α production by antigen-presenting cells (pDCs) in response to the intact IAV requires endosomal recognition of IAV genomic RNA and signaling via TLR7 and Myeloid differentiation factor 88 (MyD88) [47,48]. Lund et al. [48] demonstrated that IAVs stimulate type I IFN responses through TLR7. Feline McDonough Sarcoma (FMS)-like tyrosine kinase 3 ligand (Flt3L) cell cultures derived from TLR7–/– mice fail to produce IFN-α, IL-12 p40, and IL-6 cytokines in response to IAV genomic RNA. It has been suggested that single-stranded RNA (ssRNA) induces the critical TLR7-dependent innate responses and the influenza-derived TLR7 ligand is the viral genome [47]. RNA virus invasion upregulates type I IFN expression via TLR7 and TLR8 [49]. High TLR expression levels at antigen presenting cells are significantly associated with lower viral loads, accompanied by increased levels of signaling molecules (phospho-MAPKs, IκB) and inflammatory cytokines (IL-6, Tumor necrosis factor receptor-1(TNFR-1), chemokine (C-C motif) ligand 2 (CCL2)/ monocyte chemoattractant protein 1 (MCP-1), CXCL10/ Interferon gamma-induced protein 10 (IP-10), IFN-γ) [50]. TLR4 signaling during H5N1 infection has been reported to contribute to lung pathology [51]. The genomic RNA of IAVs is capable of inducing IFN-α response from mouse DCs in a TLR7 dependent manner [52]. TLR3 contributes directly to the immune response of respiratory epithelial cells toward double-stranded RNA (dsRNA) molecule. In a case study involving influenza patients (24 A/H3N2, 18 A/H1N1pdm09), the intracellular expression of TLR (3, 7, 8, and 9) [53] was upregulated, whereas that of TLR (2, 3, and 4) was downregulated [50]. Interestingly, IAV infection upregulates the expression of TLR3 in pulmonary epithelial cells. Mice deficient for TLR3 or its adapter molecule, toll/interleukin-1 receptor (TIR)-domain-containing adapter-inducing interferon-β (TRIF), demonstrate normal humoral and T-cell responses to sublethal influenza infection. Thus, TLR3 might be dispensable for the induction of the adaptive immune response following IAV infection [54,55], although TLR3 agonists serve as important adjuvants for influenza vaccines [56,57].

### 3.2. Retinoic Acid-Inducible Gene (RIG)-I-Like Receptors (RLRs)

The viral genome is released into the cytoplasm to initiate viral protein biosynthesis. During this step, conserved molecular structures such as triphosphates and dsRNA act as PAMPs that are recognized by sensors in the host cell cytosol [58]. To distinguish the viral genome from the host genome, RLRs comprising RIG-I, melanoma differentiation-associated protein 5 (MDA5), and other sensors such as nucleoside oligomerization domain (NOD), leucine-rich repeat (LRR), and pyrin domain (PYD) domain-containing protein 3 (NLRP3), act as intracellular viral RNA sensors [59]. Activated RIG-I signaling leads to a reduction in the antigen requirement for inducing optimal influenza-specific cellular and humoral immune responses, including protective immunity by 10- to 100-fold [60].

RIG-I and MDA5 consist of several functional domains, including two tandem amino-terminal caspase activation and recruitment domains (CARDs). The ubiquitination of K172 in the second CARD of RIG-1 is critical for IFN production in response to virus infection [61]. Tripartite motif (TRIM)25, a member of the TRIM family of IFN-inducible E3 ubiquitin ligases [61], modulates the posttranslational modification of RIG-I that alters target protein stability, trafficking, subcellular localization, enzymatic activation and protein recruitment [62]. Following ubiquitination, RIG-I initiates a signaling cascade that begins with the association of ubiquitinated CARDs of RIG-I with the CARD of the MAVS [63]. MAVS is a crucial scaffolding regulator for signal transduction [63] that recruits two signalosome complexes comprising a variety of E3 ubiquitin ligases, scaffolding proteins, and numerous protein kinases, ultimately leading to NF-kB activation [64,65].

The nonstructural protein 1 (NS1) of IAVs prevents TRIM25-mediated oligomerization to evade recognition by the host viral RNA sensor RIG-1 [66]. In addition to inhibiting IFN-β gene expression, NS1 suppresses the expression of numerous other intracellular genes involved in the RIG-I signaling cascade including the TRIM25-mediated ubiquitination [67]. A complex containing NS1, RIG-1, and possibly a viral PAMP (dsRNA), is necessary for IFN inhibition of host antiviral response.

### 3.3. Nucleotide-Binding Domain and Leucine-Rich-Repeat-Containing NLRs

Inflammasomes are caspase-1 activating platforms which include NLR family members and the apoptotic Speck protein containing a CARD (ASC) domain [68]. Caspase-1 is a key inflammatory regulator owing to its capacity to process and activate pro IL-1β, pro IL-18, and pro IL-33 [69,70]. Viral infection induces the expression of caspase-1 activating inflammasome in a cryopyrin//NACHT, LRR and PYD domains-containing protein 3 (Nalp3)-dependent manner [71]. The secretion of mature IL-1β proceeds in two sequential steps: first, up-regulation of pro–IL-1β via TLR stimulation; and second, the activation of caspase-1 by inflammasomes [72,73]. A recent report showed that influenza viruses activate the NLRP3 inflammasome in macrophages pulsed with ATP in vitro [74]. IAV triggers caspase-1 activation in wild-type (WT) but not in NLRP3−/− macrophages [75]. Mechanistically, NLRP3 inflammasomes are activated by transfection of RNA, Poly I:C, dsRNA, or ssRNA [74], and their activation by the IAV genome is dependent upon lysosomal maturation and IL-1β production. Such an inflammasomes-mediated host cell evasion mechanism may be involved in host defense against IAV infection by the viral RNA sensing. A counteracting mechanism involves the NS1 protein of IAVs containing an RNA-binding domain, which suppresses the activation of IL-1β and IL-18 secretion [76].

### 3.4. IFN Signaling

After sensing IAVs via various PRRs, the infected cells synthesize type I IFNs. In humans, type I IFN signaling is activated in response to innumerable viruses including IAVs [77]. IFNα/IFNβ binds the IFNα receptor (IFNAR), which is composed of the IFNAR1 and IFNAR2 subunits [78]. IFNAR/Janus kinases (JAK)/signal transducer and activator of transcription (STAT) signaling is augmented by immunoreceptors associated with the tonic immunoreceptor tyrosine-based activation motif (ITAM) [79]. ITAM signaling activates type I IFN-induced JAKs [80] and the phosphorylation of STAT1/STAT2. These events trigger the translocation of STAT1/STAT2 complexes into the nucleus for assembly with IFN-regulatory factor 9 (IRF9) [77]. Following translocation, the complexes bind to IFN-stimulated response elements (ISREs) and promote the activation of IFN-stimulated genes (ISG) [80]. Thus, the canonical signaling results in the transcription of hundreds of genes involved in antiviral responses [81]. Crotta et al. reported that mice deficient in type I and III IFN signaling were highly susceptible to IAV infection [82]. However, the individual deficiency of either type I or III IFN system alone produced similar transcriptional profiles of ISG, indicating that each system compensates for the loss of the other one in response to the virus [82]. The results of another study, in conjunction with the aforementioned findings on IFN signaling, suggest that type III IFN signaling also acts as the first line of defense in the pulmonary epithelium during early IAV infection, and subsequent signaling by type I IFNs may offer enhanced protection during the latter stages of viral spread [83].

## 4. Innate and Adaptive Immune Cell Response between Viral Clearance and Immunopathology

**Figure 3:**
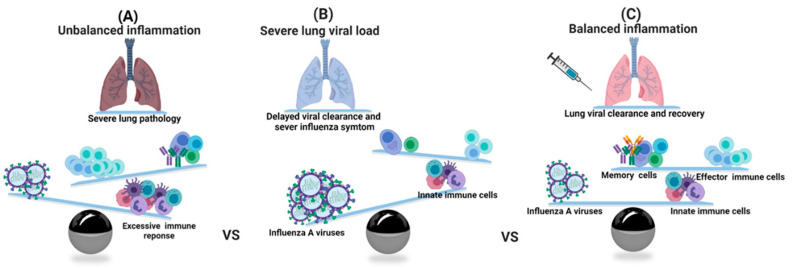
A schematic model showing the balance between successful viral clearance and a life-threatening immunopathology following influenza infection. (**A**) The excessive response to influenza infection results in the development of influenza immunopathology despite efficient viral clearance. The excessive inflammation sustained by an uncontrolled host response can induce epithelial disruption and lung damage. (**B**) Low immune response with immune escape from host immunosurveillance may increase viral replication, which in turn induces a strong release of secretory molecules. (**C**) The adequate cell mediated immunity with vaccination can control lung viral load without a severe lung pathology. This figure was created using the BioRender software.

The interplay between IAVs and the host is of pivotal importance for determining the clinical outcome of viral infection (Figure 3). Cytokines and IFNs produced during viral infection shape adaptive immune responses, including humoral and cellular immune responses. Nevertheless, in certain instances, such excessive responses are detrimental to the host. For example, infections with a highly pathogenic IAV can sometimes result in an uncontrolled and dangerous production of proinflammatory cytokines and IFNs, known as a “cytokine storm” which contributes to morbidity and mortality during the associated infection [84,85,86,87].

Endothelial cells play an essential role in leukocyte migration and early innate immune responses, particularly with respect to the production of proinflammatory cytokines and chemokines such as IL-6, monokine induced by gamma (MIG)/CXCL9, IP-10/CXCL10, type I and type II IFNs, MCP-1//CCL2, and TNF-α in response to IAV infection [88]. Upregulation of selectin and adhesion molecules for immune cell extravasation is observed in the endothelium in response to cytokines and growth factors secreted by respiratory epithelium and other neighboring cells [88]. Uncontrolled/over-activation of processes can cause more severe lung damage and further propagate inflammation (Figure 3A), rather than result in a beneficial effect by recruiting more immune cells to clear the lung viral load. Owing to the lack of pre-existing immunity against IAV, insufficient leukocyte recruitment can render the host susceptible to the virus (Figure 3B) [89]. Effective IAV vaccination can induce the expansion and proliferation of immunizing IAV-specific memory T-cells and the resulting adaptive immune responses play a major role in the resolution of subsequent infection of IAV (Figure 3C).

Neutrophils are short-lived leukocytes that rapidly migrate to the sites of infection, secrete cytokines, degranulate, and phagocytose with subsequent formation of neutrophil extracellular traps (NETs). The neutrophil trail of chemokine (C-X-C motif) ligand 12 (CXCL12) guides IAV-specific CD8+(cluster of differentiation 8) T-cell recruitment into the IVA infected tissue and enhances antiviral effector functions [90]. Neutrophils also play a substantial role in viral clearance and contribute to disease severity in the lower respiratory tract. Several studies have provided insights into the maintenance of balance between viral clearance and lung injury by interrupting chemokine ligand 2 (CXCL2; also termed macrophage inflammatory protein 2-alpha (MIP-2), growth-regulated protein beta (Gro-beta), and gro oncogene-2 (Gro-2)) or CXCL10 driven feed-forward circuits involving neutrophils [91,92,93]. Despite this, studies conducted on neutrophil depletion prior to IAV infection suggest that neutrophils are necessary for viral clearance and recovery from severe lung injury [94,95]. These data indicate a protective role of neutrophils in IAV infection; however, excessive neutrophil infiltration can also induce immunopathology [91].

Natural killer (NK) cells respond to virus-infected cells by producing significant amounts of IFN-γ, granzyme-B, and cytotoxic granules [96], engaging death receptors, and using antibody-dependent cell-mediated cytotoxicity. During both high- and low-dose IAV infection, NK cell accumulation within the lungs and airways depends on the high expression of CXCR3 and CCR5, respectively [97]. CD16− lung and peripheral blood NK cells are strongly primed after IAV infection, which contributes to host defense, but also possibly to tissue damage [98]. These results indicate the presence of a delicate balance between protective and destructive NK cell activation during various stages of IAV infection.

Tissue-resident alveolar macrophages (AMs) of the lungs are uniquely localized to the airspaces within alveoli. During steady state, AMs regularly serve as the sentinels of the respiratory tree and pulmonary mucosa. To maintain pulmonary homeostasis in response to innocuous antigens, the lung environment maintains AMs in a suppressive state, which is accomplished through the expression of IL-10, alphaV/beta6 integrin, granulocyte-macrophage colony-stimulating factor (GM-CSF), CD200 receptor, and pulmonary surfactants by the alveolar epithelium [89]. Upon IAV infection, the environment of the alveolar sac changes quickly, and the expression of the negative regulators is abolished.

As professional phagocytic cells, resident AMs eliminate infectious particles by internalization and lysosomal degradation, degradation of the dead or dying cell debris through the uptake of apoptotic cells, and facilitation of the adaptive immune response via antigen presentation to T-cells. Phagocytosis can be mediated directly via the binding of AM surface receptors to specific ligands on the phagocytic target, or indirectly via binding of fragment crystallizable gamma receptors (FcγR) of macrophage to opsonized infectious pathogens [99]. Following influenza infection, the surface expression of CD16 (cluster of differentiation 16) and CD32 on IAV infected and viral replicated macrophages decreases [100]. This may contribute to IAV pathogenesis by enhancing bacterial infection. Several reports have implicated that the lung-resident AMs are critical modulators of IAV disease severity and the development of lethal pulmonary injury [101,102,103]. This may also be critical for the antiviral antagonistic activity on Type 1 alveolar epithelial cells via AMs-mediated suppression of cysteinyl leukotriene [104].

Dendritic cells (DCs) are the most efficient antigen-presenting cells that are involved in host surveillance following IAV infection and can be activated by viruses through PRRs or proinflammatory chemokines and cytokines released by airway epithelia. Moreover, DCs activate specific T lymphocytes and promote protective adaptive immunity. In mice, resident pulmonary DCs have three major subsets including conventional CD11b+ DCs and CD103+ DCs, as well as plasmacytoid DCs (pDCs) [105]. Human pulmonary DC subsets similarly include pDCs and two subsets of myeloid DCs, the CD11b+ (CD1+ DCs) and CD103+ (CD141+/CLEC 9A DCs), that functionally resemble DC subsets observed in mice [106,107]. CD103+ acquire viral antigens and migrate to the draining lymph nodes within 2–4 days following viral infection to activate adaptive immune responses [89]. In the lymph nodes, CD103+ DCs serve as potent antigen-presenting cells for naïve CD4+ and CD8+ T-cell activation and elicit protective immunity via the presentation of viral antigens to rare virus-specific memory T-cells, which are required for adaptive immunity. Both CD103+ and CD11b+ DCs drive CD4 T-cell responses toward T helper 1 (Th1) responses and generate effective memory T-cell populations to protect against subsequent infections [108]. Unexpectedly, the lack of pDCs did not affect viral clearance or disease severity in response to IAV infection [109,110]. Owing to the low expression of costimulatory molecules, pDCs are not good as antigen-presenting cells for naïve T-cell activation and differentiation, although they can transport viral antigens from the lungs to draining lymph nodes (dLN) [89,108,111].

Effective IAV vaccination can induce the expansion and proliferation of immunizing IAV-specific memory T-cells and the resulting adaptive immune responses play a major role in the resolution of subsequent infection of IAV (Figure 3C). In the local draining LN, the antigen-carrying migratory CD103+ DCs not only present the antigens to naive T-cells but also transfer the antigens to LN-resident DCs. DCs efficiently present the antigens to T-cells, using a major histocompatibility complex (MHC) class I-binding product to prime CD8+ T-cells and an MHC class II-binding product to prime CD4+ T-cells. Functionally activated effector T-cells upregulate the levels of CCR4, MIP-1α, MCP-1, and CCL5 (RANTES; regulated upon activation, aormal T cell expressed and secreted) that guide their migration to the IAV infected airway tract [90,112]. Following migration into IAV infected lung tissue, effector CD4+ and CD8+ T-cells secrete proinflammatory cytokines (IL-12, IFN-r, and IL-2), which aid viral clearance in these cells [113]. Acute viral infection can result in the alteration of pulmonary elasticity in the terminal airways due to the organization of inflammatory processes during the resolution of the viral infection [2]. CD8+ T-cells also produce anti-inflammatory IL-10 to attenuate and resolve inflammation [114]. After the resolution of lung viral load, activated macrophages that express the co-stimulatory molecule CD86 induce the expansion of forkhead box P3 (FOXP3+) regulatory T-cells (Tregs) to promote the recovery from pulmonary diseases via the suppression of neutrophil-driven cytokine release [115].

Upon viral infection, virus-specific follicular helper (Tfh) cells facilitate the formation of germinal centers where virus-specific B-cells mature, proliferate, undergo immunoglobulin class switching, and differentiate into either antibody-secreting plasma cells or long-lived memory B-cells [116]. Virus-specific neutralizing antibodies produced by B-cells can neutralize, opsonize, inactivate virions, or initiate the killing of infected cells [89]. Neutralizing antibodies effectively prevent virus propagation by blocking surface proteins on the virus that bind to the host receptors for host cell entry [117]. Antibody-antigen immune complexes are also recognized for inactivation by complement proteins or for phagocytosis by macrophages and neutrophils [89]. Antibodies target virus or viral proteins on the surface of infected cells and then trigger the complement and antibody-dependent cell-mediated cytotoxicity (ADCC), which can eliminate antibody-coated target cells [118]. ADCC is a non-phagocytic process that requires the cooperative release of lysosomal cytotoxic granules and the expression of molecules that induce cell death mediated mainly via NK cells [119]. Although necessary to viral clearance, these excessive cytotoxic processes can impair the pulmonary function through the loss of airway epithelial cells, disruption of the integrity of the epithelia–endothelial barrier, and accumulation of apoptotic bodies containing the virus in the airways [120].

## 5. Universal Influenza Vaccine Approaches

Activation of the immune response is a necessary reaction for eliminating pathogens. A desirable vaccine is often developed by the effectiveness of the immune system and acceptable minor tissue damage associated with viral clearance. Numerous vaccine platforms against IAVs, including inactivated influenza vaccine (IIV), recombinant influenza vaccine, or live attenuated influenza vaccine (LAIV) have been licensed globally. IIVs are administered intramuscularly with trivalent or quadrivalent antigen from the influenza A (H1N1) virus, influenza A (H3N2) virus, and one or two influenza B viruses. LAIVs are approved for children and is administered via the intranasal route. A recent meta-analysis study demonstrated that the summary vaccine efficacy was 65% against any strain, 78% against matched strains, and 55% against not-matched strains [121]. The IAV vaccine efficacy is also poor in certain high-risk populations including elderly or immunocompromised patients. Passive transfer of monoclonal antibodies (mAbs) is an attractive alternative therapy for active immunization. However, this method also has drawbacks, including high costs and requirements for repeated inoculations. The vast majority of neutralizing antibodies are elicited within the HA globular head of IAVs and display strain-specific neutralizing activity [122]. However, as the globular head region of HA mutates frequently, it is necessary to reformulate and administer vaccines annually to maintain protective immunity [123]. In 2017, the National Institute of Allergy and Infectious Diseases (NIAID) identified and developed four criteria for a universal influenza vaccine: (1) at least 75% effective against symptomatic influenza virus infection, (2) protect against group I and II of IAVs, (3) produce durable protection lasting at least 1 year and preferably through multiple seasons, and (4) suitable for all ages [124]. Here, we discuss how various universal influenza vaccine strategies, which are currently undergoing clinical trials (Table 1), can play a role in the prevention of influenza.

**Figure 4:**
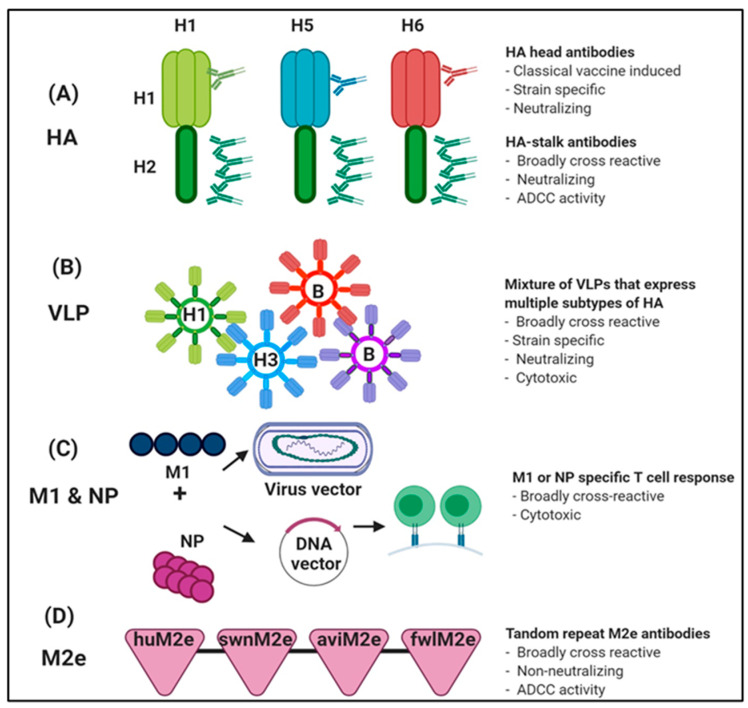
Universal IAV vaccination approaches. (**A**) Chimeric hemagglutinins (cHAs) consist of the exotic globular head domains and the conserved H2 stalk domain. (**B**) Mixture of virus like particles (VLPs) that express multiple subtypes of HA (**C**) Combination approaches with Matrix protein 1 (M1) and nucleoprotein (NP) with virus vectors or DNA vectors. (**D**) Vaccination strategies based on conserved M2 ectodomain (M2e).

The HA head domain is highly variable among IAV strains and is immunologically dominant by harboring the receptor binding sites [125]. The stalk domain of HA that is relatively conserved between different subtypes of IAV and elicits stalk specific antibodies is key for the development of a universal influenza vaccine (UIV). The importance of the first results of the stalk-based vaccines that were cross-reactive among IAVs [126] was not immediately recognized. While strategies involving the conserved stalk regions have demonstrated a broad spectrum of protection against various influenza subtypes in animal models, the protective efficacy of HA-stalk-based vaccines is relatively weak. Multiple boost immunizations are required for sufficient protection due to the low immunogenicity of the domain [127,128]. Recently, the chimeric HA (cHA) approach involving fusion with stalk domains of H1, H3, or B, and the exotic globular head domains of H5, H6, or H8 showed a full-length functional HA protein [129,130,131]. In a preclinical study, ferrets that received a sequential immunization with heterologous influenza strains, including LAIV bearing an H8 head domain and an H1 stem domain (cH8/1) and a split-inactivated vaccine bearing an H5 head domain and an H1 stem domain (cH5/1), demonstrated superior protection when challenged with pandemic H1N1 virus following different prime-boost combinations and immunization regimens (Figure 4A) [132]. This prime/boost approach with cHA has been tested in phase I clinical study [133]. The approach involving the hyperglycosylation of the globular head domain of HA redirected humoral response toward the conserved stalk region and provided better protection than the WT HA in a mouse model [134].

As an attractive vaccine delivery system, virus-like particles (VLPs) demonstrating a deletion mutant of headless HA [135] and nanoparticle structures consisting of the stalk antigens [136,137] show a broadly protective efficacy in animal models. Additionally, a variety of VLP approaches have been used for IAV vaccination, including VLPs bearing both HA and NA [138], VLPs bearing either HA or NA [139,140] or VLPs bearing more than one HA (Figure 4B) [141,142]. Recent results of the vaccination with a mixture of VLPs that individually display H1, H3, H5, or H7 Has have been promising [143]. Recently, plant-based quadrivalent virus-like particle (QVLP) vaccines have been extensively undergoing clinical trials for testing efficacy, safety, and immunogenicity of VLPs in humans [144,145,146,147,148]. The edible oral vaccines format will provide an alternative vaccine platform with needle-free, non-invasive, and cost-effective advantages for developing and low-income countries in the near future.

The M1 and NP are both important structural proteins of the virus that are highly conserved across subtypes of IAV (Figure 4C). M1 and NP are located inside the virus or infected cells and cannot be readily recognized by antibodies [149]. However, such internal viral proteins exhibit significant expression of cytotoxic CD8 T cell epitopes and can provide protective immunity against IAV infection [150]. In healthy human subjects, M1- and NP-specific memory CD4+ and CD8+ T cells generated by IAV infection highly correlate with protection in adults [151,152] and the elderly population [153]. In addition to these viral vectored vaccines expressing conserved NP and M1 antigens, the bivalent viral vectors expressing the fusion proteins (chimeric HA or NP+M1) confer broad protection against homologous and drifted viruses in mouse models [154]. Recently completed clinical trials based on prime/boost regimens employing viral vectored vaccines with NP + M1 (viral vectored vaccine based on Modified Vaccinia virus Ankara (MVA) and Chimpanzee Adenovirus Oxford 1 (ChAdOx1) expressing influenza NP and M1 proteins) induced T-cell mediated immune responses [155]. Broad protection induced by efficient influenza antigens is associated with viral clearance mediated by broadly reactive cytotoxic CD8+ T cells, reducing the severity of clinical pathogenic outcomes [156]. Other candidates for universal vaccine strategy include multi-valent synthetic peptides exhibiting a strong CD8+ T cell epitope of the NP, M1, M2, and PB1 [157].

M1 and M2 protein are universal molecules present in influenza viruses with different strains of hemagglutinins and neuraminidases. These proteins were discovered nearly four decades ago [158]. Recently, less variable virus structures, such as the extracellular domain of the M2 protein (M2e), have also been considered to be a promising target for eliciting the expression of broadly reactive antibodies and for potent cross-protection (Figure 4D). M2e is a highly conservative region in various subtypes of IAVs. This region is highly expressed on infected cells [149,159].However, approaches that target M2e require a delivery system to enhance immunogenicity and protective efficacy because of its weak immunogenicity [160]. Furthermore, M2e and influenza virus nucleoprotein (M2eNP) DNA vaccination of pigs exacerbated influenza disease after challenge with the lethal swine subtype of IAV [161]. Many promising preclinical trials have not yet been translated into clinical trials or approved for human use because of a bottleneck to preclinical development, limitations of adequate animal models, or requirement of industry funding for the increasing scale of clinical studies [11].

Nonetheless, owing to the tetrameric structure of M2e consisting of two subunits linked by a disulfide bond [162], it has been suggested that tandem repeated M2e multiple domains could be used in the VLP form [163,164,165]. Several VLP vaccines expressing M2e have been studied to improve protection against IAVs with various cores of VLPs including hepatitis B virus [166], Papaya mosaic virus [167], Malva mosaic virus [168], tobacco mosaic virus [169], -core proteins, and the influenza matrix protein-transmembrane (TM) domain of HA [170,171] fused with M2e. Alternatively, such attractive targets for the construction of the universal vaccine can be used as scaffolds to expose severe acute respiratory syndrome coronavirus 2 (SARS-CoV-2) antigens and generate a bivalent vaccine targeting two relevant pathogens causing respiratory diseases [172]. These novel approaches using universal influenza virus vaccines have potential advantages such as the termination of the requirement of annual re-formulation and re-administration of influenza vaccines [149]. For the rapid development and availability of universal influenza vaccines, the UIV requires the identification and standardization of defined points of protective immune correlates, and consideration of a dosage schedule to maximize vaccine uptake [11,173,174]. The universal influenza vaccine technologies will pave the way for the development of improved and effective universal vaccines against future emerging viruses.

## 6. Conclusions

IAVs have evolved multiple strategies to counter the “first line of defense” embodied by the host immune system to replicate efficiently. Under homeostatic conditions, balanced immune responses are fine-tuned by host factors at multiple levels, interacting with many transcription factors and regulatory proteins, which are involved in host defense and survival. A broad spectrum of viral sensors in the host respiratory epithelium have evolved multi-layered defenses against IAVs. Upon infection with an IAV, these sequential reactions essentially result in the antiviral states, whereas dysregulated immune reactions can cause serious pathogenesis. Comprehensive knowledge of IAV-host interactions is needed to develop clinically useful prophylactic targets and the molecular mechanisms of viral pathogenesis to combat IAV infection. Future universal IAV vaccine platform should include not only the HA and NA antigens, but also the highly conserved stem of HA, NA, NP, and M2 antigens to enhance broad cross-protection, heterosubtypic immunity, and long-lasting protection against annually updated IAVs. To provide broadly protective universal vaccines against IAV, many groups are evaluating universal vaccines by using distinct platforms and strategies in healthy or high-risk populations. Their findings can serve as a template for potential strategies for improved vaccines against seasonal influenza and newly emerging viruses.

## Figures and Tables

**Table 1:** Vaccine candidates currently being developed for the universal influenza vaccine under clinical trials. Update; 11 September 2020.

Study Title	Sponsor	Phase/Ages/Route	Strategies or Formulation	National Clinical Trial (NCT) ID
A Study to Evaluate the Reactogenicity, Safety, and Immunogenicity of Investigational Supra-seasonal Universal Influenza Vaccines from GlaxoSmithKline Biologicals- Inactivated (GSK3816302A)	GlaxoSmithKline	Phase I/18–39/IM	Investigational supra-seasonal universal influenza vaccines (SUIVs) of Biologicals supra-seasonal universal influenza vaccines (SUIVs)	NCT03275389
Safety and Immunogenicity of a Live-attenuated Universal Flu Vaccine Followed by an Inactivated Universal Flu Vaccine	PATH	Phase I/18–39/nostril	Prime- boost regimen: Live attenuated influenza vaccine (LAIV) cH8/1N1 prime and inactivated split influenza vaccine (IIV) cH5/1N1 cH8/1N1 boost	NCT03300050
Immunogenicity and Safety Study of Inactivated Subunit H5N1 Influenza Vaccine in Prior Recipients of Live Attenuated H2N2, H6N1, and H9N2 Influenza Vaccines and in H5N1 and Live Attenuated Vaccine Naïve Individuals	National Institute of Allergy and Infectious Diseases (NIAID)	Phase I/18–60/IM	Prime-Boost approach: Pandemic live attenuated influenza vaccines (pLAIVs) H2N2, H6N1, or H9N2 prime and pandemic inactivated subunit vaccine H5N1 pandemic inactivated subunit vaccine (pISV) boost	NCT03816878
A Pivotal Trial to Assess the Safety and Clinical Efficacy of the M-001 as a Standalone Universal Flu Vaccine	BiondVax Pharmaceuticals Ltd.	Phase III/50–64 and over 65/IM	Multimeric-001: A recombinant protein containing 9 conserved epitopes from Influenza A and B	NCT03450915
A Phase I Study of Candidate Influenza Vaccines MVA-NP+M1 and ChAdOx1 NP+M1	University of Oxford	Phase I/18–/IM	MVA consists of the complete NP and M1 from A/Panama/2007/99 joined by a 7 amino acid linker sequence and is expressed from the Vaccinia P7.5 promoter inserted at the thymidine kinase locus of MVA	NCT01818362
Study of VGX-3400X, H5N1 Avian Influenza Virus DNA Plasmid + Electroporation in Healthy Adults	Inovio Pharmaceuticals	Phase I/18–50/IM-EP (Intramuscular injection followed by electroporation)	DNA Plasmid Vaccine for H5N1 Avian Influenza (VGX-3400X)	NCT01142362
A Randomized, Double-blind, Placebo-controlled Phase IIb Trial to Test FLU-v Vaccine	PepTcell Limited	Phase II/18–60/Subcutaneous	FLU-v(synthetic multiepitope peptides) is a sterile equimolar mixture of four synthetic polypeptides: M1 (32 aa), NPA; nucleoproteins A (21 aa), NPB; nucleoproteins B (20 aa), M2 (24 aa)	NCT02962908
Efficacy of a Plant-derived Quadrivalent VLP Vaccine in the Elderly	Medicago	Phase III/65 and older/IM	Quadrivalent VLP influenza vaccine: a mix of recombinant H1, H3, and two B hemagglutinin proteins expressed as VLPs for the 2018–2019 influenza virus strains	NCT03739112
Dose, Safety, Tolerability and Immunogenicity of an Influenza H1 Stabilized Stem Ferritin Vaccine, VRCFLUNPF099-00-VP, in Healthy Adults	NIAID	Phase I/18–70/IM	Biological: VRC-FLUNPF099-00-VP (H1ssF_3928) The vaccine is composed of the HA stem domain from Influenza A/New Caledonia/20/l999 (HlNl) genetically fused to the ferritin protein from H. pylori. Purified HlssF_3928 displays eight well-formed HA trimers that antigenically resemble the native Hl stem viral spikes.	NCT03814720
Safety, Tolerability, and Immunogenicity of VAL-339851 (mRNA-1851) in Healthy Adult Subjects	ModernaTX, Inc.	Phase I/18–49/IM	Biological: VAL-339851 modified mRNA/lipid vaccines against H10N8 and H7N9 influenza viruses. H10N8 intramuscular (IM) dose levels of 25, 50, 75, 100, and 400 µg and intradermal dose levels of 25 and 50 µg. H7N9 IM 10-, 25-, and 50-µg dose levels	NCT03345043
